# A Comparative Analysis of *Gremlin-1 (GREM1)*, *Hyaluronic Acid Synthetase-2 (HAS2)*, and *Prostaglandin-Endoperoxide Synthase-2 (PTGS2)* Expression in Cumulus Cells Among Women with Diminished Ovarian Reserve Following Rescue In Vitro Maturation (r-IVM)

**DOI:** 10.3390/life15101609

**Published:** 2025-10-16

**Authors:** Mohd Faizal Ahmad, Marjanu Hikmah Elias, Norazilah Mat Jin, Muhammad Azrai Abu, Saiful Effendi Syafruddin, Ani Amelia Zainuddin, Shah Shamsul Azhar, Nao Suzuki, Abdul Kadir Abdul Karim

**Affiliations:** 1 Advanced Reproductive Centre (ARC) HCTM UKM, Department of Obstetrics & Gynecology, Faculty of Medicine, National University of Malaysia, Jalan Yaacob Latiff, Bandar Tun Razak, Kuala Lumpur 56000, Malaysia; drmohdfaizal@ukm.edu.my (M.F.A.); azraiabu1983@gmail.com (M.A.A.); aniameliaz71@gmail.com (A.A.Z.); 2 Faculty of Medicine & Health Sciences, Universiti Sains Islam Malaysia, Nilai 71800, Negeri Sembilan, Malaysia; marjanuhikmah@usim.edu.my; 3 Department of Obstetrics & Gynecology, Faculty of Medicine, Universiti Teknologi MARA, Sungai Buloh Campus, Selangor Branch, Jalan Hospital, Sungai Buloh 47000, Selangor, Malaysia; drnorazilah@yahoo.com; 4 Medical Molecular Biology Institute, National University of Malaysia, Jalan Yaacob Latiff, Bandar Tun Razak, Kuala Lumpur 56000, Malaysia; effendisy@ppukm.ukm.edu.my; 5 Department of Public Health, Faculty of Medicine, Universiti Kebangsaan Malaysia, Cheras, Kuala Lumpur 56000, Malaysia; drsham@ppukm.ukm.edu.my; 6 Department of Obstetrics & Gynecology, St. Marianna School of Medicine, Kawasaki 216-8511, Kanagawa, Japan; nao@marianna-u.ac.jp

**Keywords:** *HAS2*, *PTGS2*, *GREM1*, oocyte quality, diminished ovarian reserve

## Abstract

Managing women with diminished ovarian reserve for in vitro fertilization (IVF) is challenging, often resulting in low oocyte yield and cycle failures. We hypothesize that coupling in vitro fertilization (IVF) with rescue in vitro maturation (r-IVM) can improve overall maturation rates without compromising the overall IVF outcome. Our study compared DOR and normal ovarian reserve (NOR) cohorts by evaluating 15 immature oocyte progressions following r-IVM. We analyzed the gene expression of cumulus cells related to *GREM1*, *PTGS2*, and *HAS2* to correlate with OQ, EQ, and overall IVF outcome. Significant differences were noted in AMH levels, AFCs, and oocyte numbers (*p* < 0.05). Following r-IVM, the DOR cohort achieved a 50% maturation rate with improved overall quality; however, the difference was not statistically significant (*p* > 0.05). Fertilization rates were comparable, but EQ was better in DOR. All genes in DOR were upregulated post-r-IVM, whereas NOR showed downregulation of *PTGS2* and *GREM1* (*p* < 0.05). Otherwise, DOR exhibited higher pregnancy rates and live birth rates, although the difference was not statistically significant (*p* > 0.05). Overall, our findings suggest that r-IVM could provide improved fertility outcomes for DOR women in standard IVF cycles.

## 1. Introduction

In vitro maturation of oocytes is not a new concept. It was first implemented in 1960 using an animal model and has since evolved significantly for use in the human reproductive field, resulting in the first in Vitro Maturation (IVM) pregnancy and successful delivery [1,2,3]. Since then, IVM has been incorporated with standard in vitro fertilization (IVF) for improved oocyte maturation and embryo yield in the targeted population, mainly in individuals with polycystic ovarian syndrome (PCOS) [3]. Physiologically, IVM requires incubation of the cumulus–oocyte complex (COC) in specialized media consisting of follicular-stimulating hormone (FSH), luteinizing hormone (LH), and other supporting components, such as growth factors and albumin [4]. The interaction of COCs with media competence is crucial to achieve comparable maturation in vivo. Various groups established their own IVM regime preferences depending on the recommended protocol.

To date, the standard IVM has been confined to standard stimulation without a trigger agent, mainly LH or hCG, prior to oocyte retrieval (OR) [3,5,6]. The latter is considered fundamental to ensure that the COC remains quiescent, mainly to prevent the breakdown of a germinal vesicle (GV) and the subsequent activation of the LH receptor (LHR), which might activate the signaling pathway; it leads to the expulsion of the first polar body [7], causing premature oocyte maturation and poor IVM outcome. Therefore, most of the studies on implementation adopt the standard IVM as an acceptable clinical practice [8,9,10]. Nevertheless, as the standard IVM outcome is still inconclusive with various oocyte maturation rates (OMR) reported worldwide, the reinforcement of IVM culture was then introduced as an initial step prior to IVM culture, namely, capacitation (CAPA)-IVM, which requires a two-step culture to ensure improved results [11]. To date, CAPA-IVM is the most established IVM strategy, mainly for women with PCOS [12]. Concerning the IVM protocol, utilizing LH triggers prior to IVM culture is still being opted for and considered non-standard IVM [3,8].

**Figure 1:**
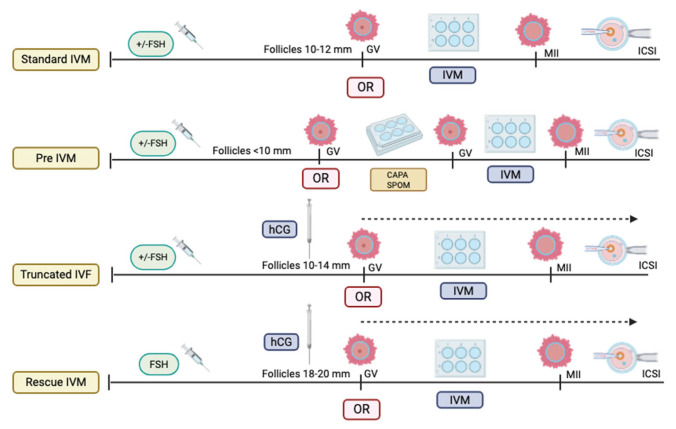
Types of in vitro maturation (IVM) protocol (Created by https://BioRender.com/otdzckl accessed on 10 May 2025).

**Figure 2:**
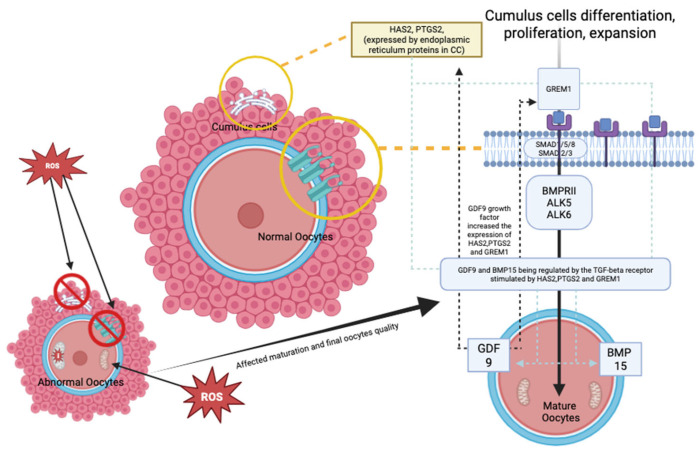
Oocyte maturation mechanism—comparing normal and abnormal oocytes (Created by https://BioRender.com/otdzckl accessed on 10 May 2025).

Most published evidence suggests that the outcome is comparable to that of standard IVM; thus, the role of LH in IVM protocols remains inconclusive, depending on the center. In addition, in the stimulated cycle, the LH trigger is often given to ensure the initiation of maturation in vivo, and the rest of the maturation can be continued in vitro. Most of these cycles require OR once the dominant follicles reach 12–14 mm. In contrast to the non-stimulated cycle, the LH trigger is often omitted prior to the OR procedure, in which the follicles are only at 10–12 mm [3]. Thus, the IVM protocol is currently not standardized worldwide [8,10]. All the current IVM protocols are illustrated in Figure 1. On the other hand, emerging rescue-IVM (r-IVM) has also been reported to be part of the current IVM protocol [13]. It is complemented by standard IVF, which harvests dominant follicles of size 18–20 mm, and if there are immature oocytes, r-IVM will be offered. Therefore, r-IVM utilizes a full dose of gonadotropin to ensure the complete optimization of follicle development and requires an LH trigger prior to OR. However, the r-IVM outcome was reported as not favorable [14,15]. Hence, it was not widely practiced. Most centers opted for r-IVM for a standard cohort of women for immature oocytes following OR [14]. These immature in vivo oocytes were reported as molecularly abnormal; thus, not surprisingly, the outcome is not acceptable despite r-IVM [16]. The COC provides a vital network for maintaining the molecular environment necessary for cell differentiation, proliferation, and expansion [17,18,19]. The TGF-beta receptor plays a key role in regulating GDF-9 in conjunction with the BMP15 family, impacting the expression of essential genes, particularly *HAS2* and *PTGS2*, through endoplasmic reticulum proteins in cumulus cells (CCs) and *GREM1* via receptor phosphorylation [20,21]. This process is crucial for the maturation of oocytes and for maintaining overall competency. However, in abnormal oocytes with high reactive oxidative stress due to any factor, the COC will be dysfunctional, leading to poor oocyte development and low oocyte competency [22] (Figure 2). Such issues are frequently observed in aging populations, DOR women, women with endometriosis, those with PCOS, and women undergoing oncofertility treatments following chemotherapy [23]. Furthermore, most cases of r-IVM were conducted following the denudation of oocytes to break the vital COC for IVM [14]. Therefore, OMR was reported to be low, and impaired fertilization rates (FR), poor overall oocyte quality [24], and embryo quality (EQ) were observed following r-IVM [25]. Evidence indicates that r-IVM among individuals with DOR leads to a high yield of utilized oocytes and embryos following the IVF cycle [5,26,27]. Most immature oocytes from women with DOR can mature in vitro through r-IVM. Many of these oocytes could not respond to the LH trigger in vivo due to suboptimal signaling within the follicular microenvironment. After ovarian reserve (OR) assessment, retrieval rates with r-IVM were reported to be at least 30–50%, along with improved OQ and EQ [5,28,29]. Oocytes from women with normal ovarian reserve (NOR) can be molecularly abnormal [30,31]. Therefore, if maturation in vivo is unsuccessful, then concerns about the effectiveness of IVM culture are raised. The primary concern is the OQ alteration following r-IVM, which leads to an overall poor IVF outcome [32,33]. The selected cohort for r-IVM is primarily recommended for women with diminished ovarian reserve [34] or those who are older [29]. However, limited evidence is available regarding r-IVM in this group. In this regard, the present study aimed to evaluate the overall OQ in DOR women undergoing r-IVM and correlate it with EQ and pregnancy outcomes. Therefore, in our study, we used established gene expression markers for OQ assessment, specifically examining the expression levels of GREM1, HAS2, and PTGS2 in CCs before and after r-IVM compared to the NOR cohort. Our r-IVM process involved using intact COCs without denudation to preserve their integrity during the procedure. In addition to assessing OQ and EQ, we also investigated overall IVF outcomes following r-IVM in women with DOR compared to those who underwent standard procedures. Our findings strengthen existing evidence for r-IVM in DOR and establish it as a viable strategy for future IVF treatments.

## 2. Materials and Methods

### 2.1. Study Design

This prospective study was performed from July 2022 to July 2023 at the Advanced Reproductive Center (ARC), Hospital Canselor Tuanku Mukhriz (HCTM) UKM, Cheras Kuala Lumpur, Malaysia. Our research received approval from the Human Ethical Research Committee of the Faculty of Medicine at the National University of Malaysia, with registration numbers JEP-2023-360 and JEP-2022-187. Before recruitment, we obtained informed and verbal consent from all participants. Based on the Bologna criteria, the study cohort was classified into two groups: the NOR cohort and the DOR cohort. A serum anti-Müllerian hormone (AMH) level of less than 1.2 ng/mL and an antral follicle count (AFC) of fewer than five on initial transvaginal ultrasound (TVS) screening during day two or three of the menstrual cycle indicated DOR [35]. All women in the study underwent controlled ovarian hyperstimulation (COH) for IVF during the study period by utilizing an antagonist protocol. The choice of gonadotropin was determined by the clinicians’ preference, with the option of combining it with or without oral agents, such as clomiphene citrate, an aromatase inhibitor, and trigger agents.

### 2.2. COH, r-IVM, IVF Protocol, and CC Collection

Our standard COH protocol involves using a combination of gonadotropins, specifically either Folliculin^®^ (BSV, Mumbai, India) or follitropin alfa (Gonaf F^®^, Merck, Darmstadt, Germany), alongside highly purified menotrophin (Humog^®^ BSV, Mumbai, India). The initial combination dose is 225 IU daily, which can be gradually increased to 375 IU based on the response of follicular growth. An additional antagonist injection, either Cetrotide^®^ (Merck, Darmstadt, Germany) or Asporelix^®^ (BSV, Mumbai, India), is administered once the follicles reach a size of 10 mm to 12 mm to prevent premature ovulation. Our minimal stimulation protocol consists of oral ovulation induction by using either an aromatase inhibitor or clomiphene citrate, which is introduced during the first five days. This is accompanied by highly purified menotrophin (Humog^®^ BSV, Mumbai, India) at a dosage of 225 IU on alternating days (specifically on days 3, 5, and 9) during ovarian stimulation. An additional injection of antagonists, such as Cetrotide^®^ (Merck, Darmstadt, Germany) or Asporelix^®^ (BSV, Mumbai, India), is given once the follicles reach 10 mm to 12 mm to prevent premature ovulation. Minimal stimulation is preferred in the DOR cohort because it is believed to improve oocyte quality, even though it decreases the number of oocytes, total gonadotropin dose, and cost [36]. Our center adopts a minimal stimulation protocol as standard practice, depending on the clinician’s preferences.

In addition to that, in our practice, once two or more dominant follicles [37] reached a mature size of 18–20 mm, the triggering agent (Lh/hCG) was administered. The agent could be Ovidrel^®^ (Merck, Darmstadt, Germany), Hucog^®^ (BSV, Mumbai, India), or Decapeptyl^®^ (Ferring, Saint-Prex, Switzerland), depending on the clinician’s preference. A dual trigger comprising the options mentioned above could also be used based on the clinician’s decision. Subsequently, OR was then performed at least 34–36 h after the triggering agent was administered under sedation according to local protocols. The OR procedure was performed as a standard procedure using a double-lumen needle (Cook^®^, Bloomington, IN, USA) with a pressure of 120–140 mmHg. Mechanical oocyte denudation (OD) was gently performed using only manual force generated by repetitive pipette aspiration and deposition in a series of media to assess maturation without enzymatic incubation, utilizing Hyase^®^ (Vitrolife Sweden AB, Göteborg, Sweden) to minimize damage to the COC and, most importantly, to prevent CC lysis and RNA degradation. All mature oocytes identified as being in metaphase II (MII) were then incubated with Hyase^®^ (Vitrolife Sweden AB) for proper denudation and subsequent intracytoplasmic sperm injection (ICSI). Conversely, immature oocytes identified as germinal vesicle (GV) or metaphase I (MI) were incubated with IVM media (Kitazato^®^, Tokyo, Japan) for up to 48 h as part of the r-IVM procedure. Specifically, for MI oocytes, a 6–12 h check was performed, and incubation could be extended up to 24 to 48 h. Prior to incubation, CCs were collected for RNA extraction and cDNA synthesis to evaluate gene expression before r-IVM. After r-IVM, all possible mature COCs were denuded, and CCs were again collected for RNA extraction and cDNA synthesis to assess gene expression following r-IVM. All samples collected were kept in a 1.5 mL Eppendorf tube filled with 500 µL RNALater^®^ solution (Thermo Fisher, Waltham, MA, USA) and placed at −80 °C for RNA extraction. All successful mature MII oocytes were incubated with Hyase^®^ for appropriate denudation before proceeding to ICSI, while MI and GV oocytes were discarded.

### 2.3. RNA Extraction, cDNA Synthesis, and qPCR

RNA extraction, cDNA synthesis, and qPCR protocol were conducted following the manufacturer’s instructions (Qiagen^®^, Germantown, MD, USA). In brief, the CCs were homogenized with BioMasher III (Funakoshi^®^, Tokyo, Japan). The CCs were separated from the RNA stabilizer through 2000× *g* centrifugation for 10 s, and the filtrate was discarded. To the filter column was added 100 µL of lysis buffer, and the CCs were ground using the pestle provided. Additionally, 100 µL of lysis buffer was added, and the disrupted samples were homogenized through the filter using centrifugation at 10,000× *g* for 30 s. The “pass-through” homogenized samples were collected for RNA purification using an RNAeasy^®^ microkit (Qiagen) following the manufacturer’s protocol. During the final elution, a new 1.5 mL microcentrifuge tube was placed on the RNAeasy column, and 14 µL RNAse-free water was added at the direct center of the RNAeasy column and set for 5 min. The lid was closed gently, and centrifugation was performed at full speed for 1 min to obtain the final RNA. The concentration and purity of the purified RNAs were measured using a Nanodrop. The purity of the samples was evaluated by measuring the ratio of absorbance readings at 260 nm (specific for nucleic acids). Samples with A260/A280 ratios between 1.80 and 2.10 were considered to have no significant RNA contamination [38]. The ratio A260/A230 was used as the secondary measure of nucleic acid purity and should be within 2.0–2.2.

**Figure 3:**
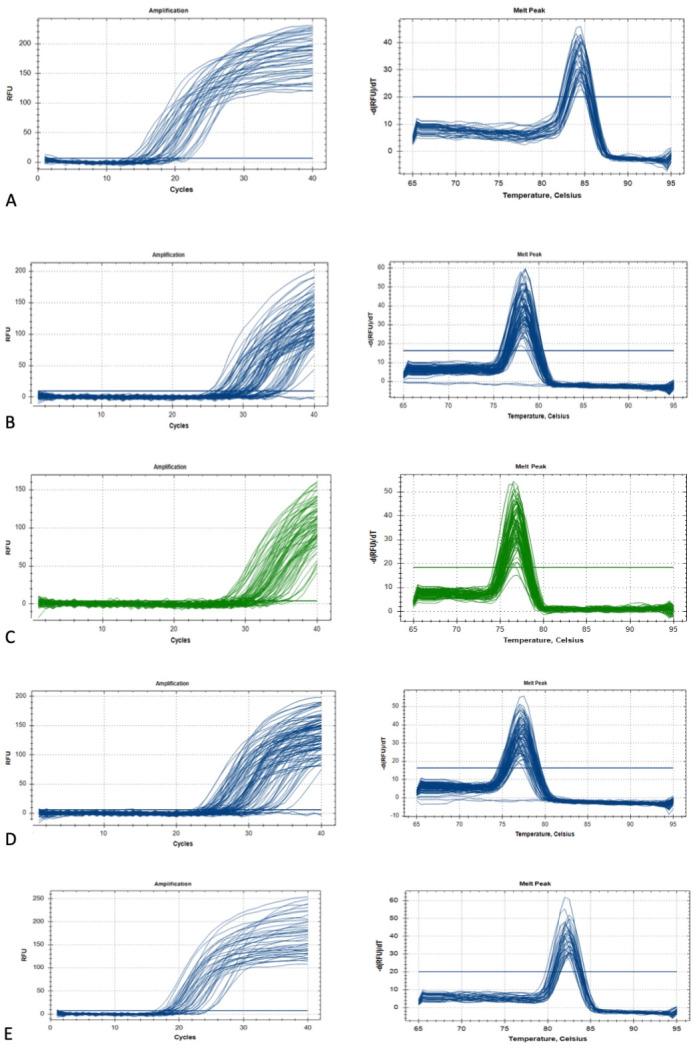
The GOI and HK gene amplification and melting curve: (**A**) GAPDH, (**B**) GREM1, (**C**) HAS2, (**D**) PTGS2, and (**E**) RR18S.

**Figure 4:**
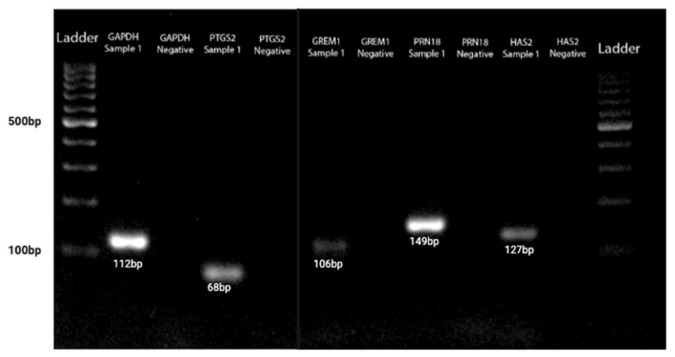
The GOI and HK genes agarose gel electrophoresis acid based on DNA fragments based on their base pair size.

The QuantiTect reverse transcription kit (Qiagen^®^, USA) was used for cDNA synthesis. First, genomic DNA elimination was carried out on ice, with a total volume of 14 µL, and incubated for 2 min at 42 °C. The master mix solution was prepared according to the protocol, resulting in a final volume of 20 µL, which included 14 µL of template RNA. This solution was then incubated for 15 min at 42 °C, followed by 3 min at 95 °C to inactivate Quantiscript reverse transcriptase. Quantitative PCR (qPCR) amplifications were conducted using the RT^2^ SYBR Green qPCR Mastermix (Qiagen^®^, USA). Two reference genes, 18S ribosomal RNA (*RRN18S*) and *GAPDH*, were used for cDNA amplification. The primer sequences Hs_GAPDH_vb.1_SG and Hs_RRN18S_1_SG from the QuantiTect Primer Assay (Qiagen^®^, USA) were employed for qPCR amplification. Additionally, genes of interest (GOI), including *GREM1* (Hs_GREM1_1_SG), *HAS2* (Hs_HAS2_1_SG), and *PTGS2* (Hs_PTGS2_1_SG) from the same QuantiTect Primer Assay were amplified. The qPCR master mix contained 1× RT^2^ SYBR Green qPCR Mastermix (Qiagen^®^, USA), primers at a concentration of 1 pmol/µL, 3 µL of cDNA, and deionized distilled water added to a total volume of 20 µL. Amplifications were performed using the CFX96™ Real-Time PCR Detection System (Bio-Rad, Hercules, CA, USA) following this cycle protocol: 95 °C for 15 min, followed by 39 cycles of 94 °C for 15 s, 60 °C for 30 s, and 72 °C for 30 s. A melt curve analysis was conducted according to standard settings (Figure 3), and agarose gel electrophoresis was then performed to analyze DNA fragments based on their base pair size, as per the manufacturer (Figure 4).

### 2.4. Oocyte Quality [2] Assessment and Embryological Outcome

**Figure 5:**
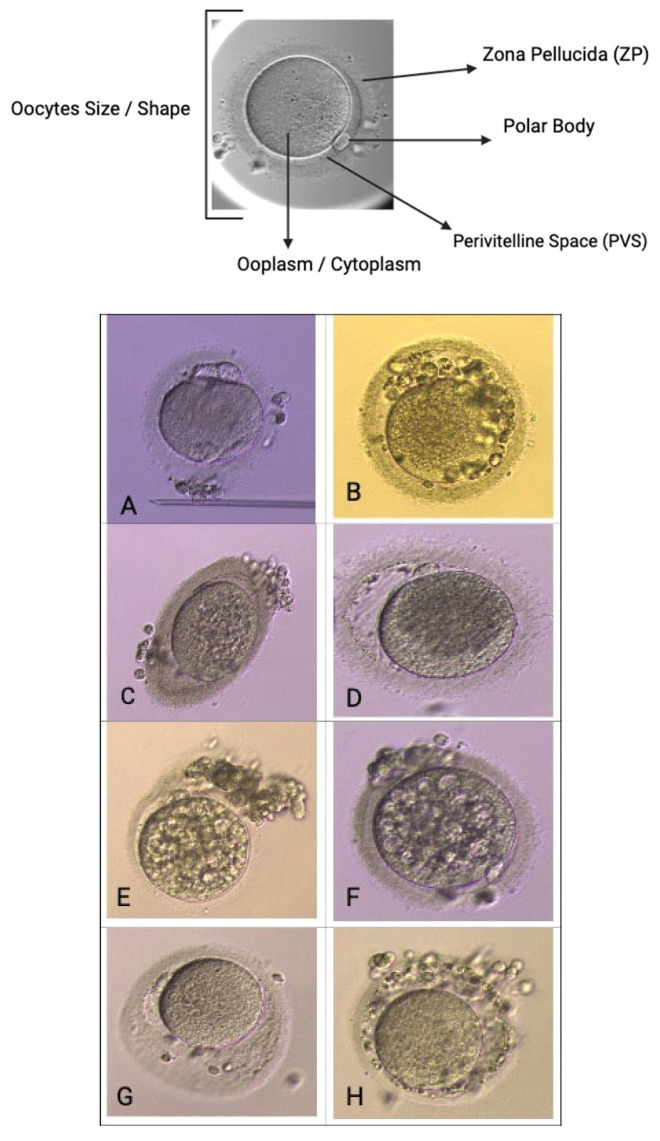
Oocyte quality assessment via morphology. Scoring base on size/shape, Zona Pellucida (ZP), polar body [7], perivitelline space (pvs), ooplasm/cytoplasm. (**A**)—abnormal PB, cytoplasm, shape, (**B**)—abnormal PVS and cytoplasm, multiple PB, (**C**)—oval shape with abnormal cytoplasm, (**D**)—large PVS and coarse cytoplasm, (**E**,**F**)—multiple vacuoles cytoplasm, (**G**)—large, abnormal PVS, (**H**)—coarse ZP and abnormal PVS.

**Figure 6:**
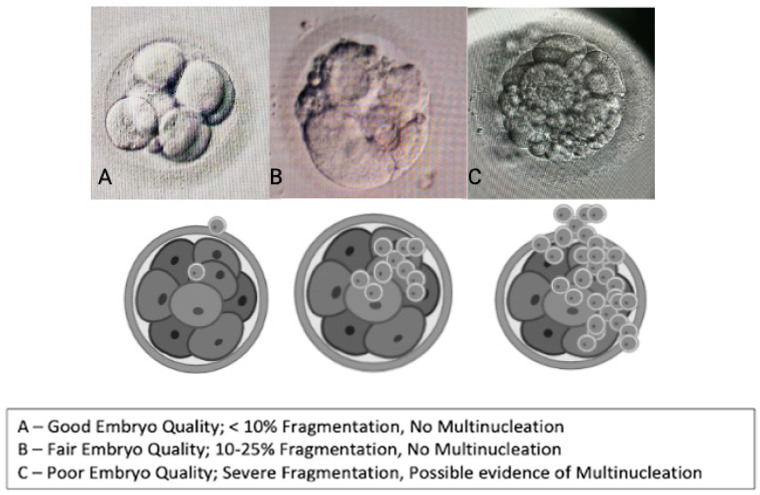
Embryo quality scoring via morphology (cleavage stage—D3).

**Figure 7:**
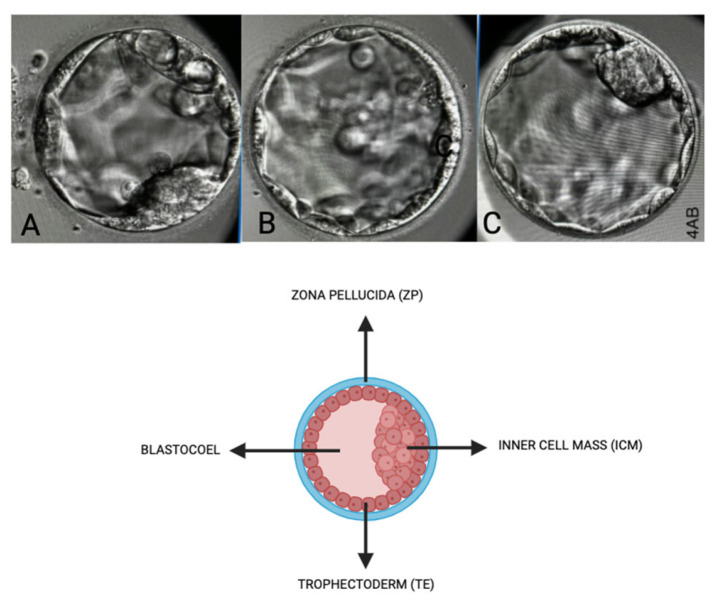
Embryo quality scoring via morphology (blastocyst stage—D5). Scoring based on inner cell mass (ICM), Zona Pellucida (ZP), Trophectoderm (TE), and Blastocoel. (**A**)—Score: 4BC, (**B**)—Score: 4CB, (**C**)—Score: 4AA.

Oocyte quality assessment was performed using morphology scoring based on the method described by Wang Q et al. and further adapted to our local protocol. In our laboratory, oocytes were evaluated according to their overall structures, which include the polar body [7], ooplasm/cytoplasm, zona pellucida (ZP), and perivitelline space (PVS) [39,40]. We also assessed other abnormalities that may affect the oocyte quality, such as vacuoles, smooth endoplasmic reticulum (SER) aggregation, and lipofuscin or refractile inclusion bodies. Oocytes were graded based on the number of abnormalities observed: those with 1–2 abnormalities are classified as “good,” those with 3–4 abnormalities as “fair,” and those with 5–6 abnormalities or severe issues (including large vacuoles, SER aggregation, or large inclusion bodies greater than 5 µm) are classified as “poor” (Figure 5). Regarding embryological outcomes, our primary focus was on the oocyte maturation rate (OMR), which was defined as the ratio of mature oocytes (MII) to immature oocytes cultured. Our secondary outcomes included fertilization rate (FR), which was calculated as the percentage of micro-injected MII oocytes that developed into two pronuclei (2PN). Additionally, the cleavage and blastocyst rates were assessed by counting the total number of day 3 (D3) embryos and day 5 (D5) blastocysts concerning the total number of fertilized oocytes. Our center, D3 EQ, was evaluated according to the ESHRE Istanbul Consensus [7]. In this framework, good embryo quality was characterized by less than 10% fragmentation and the absence of multinucleation. Fair embryo quality was indicated by 10% to 25% fragmentation without multinucleation. Poor embryo quality was defined by severe fragmentation, which may occur alongside multinucleation (Figure 6). For scoring D5 blastocysts, we utilized a modified version of the Gardner scoring system tailored to our local protocol [41,42]. This scoring system evaluated cellular expansion as 1 to 6, with six indicating the most expanded (6—blastocoel 100% with fully hatched; 5—blastocoel 100% with hatching; 4—ZP thin with blastocoel 100%; 3—ZP thick with blastocoel 100%; 2—ZP thick blastocoel >50%; 1—ZP thick with blastocoel <50%), whereas the quality of the inner cell mass was rated A to C, with A representing the best quality (A: Good—tight packed ICM with many cells; B: Fair—loosely grouped ICM with several cells; C: Poor—very few cells and disorganized). The quality of the trophectoderm was also rated A to C, with A indicating the best quality (A—TE with many cells with forming cohesive epithelium; B—few cells forming a loose epithelium; C—very few large cells) (Figure 7).

### 2.5. Pregnancy and Live Birth Outcome

Secondary outcomes included pregnancy and live birth rates. Clinical pregnancy is defined as having a positive beta-hCG level accompanied by the presence of a gestational sac confirmed through ultrasound (USG). Chemical pregnancy is identified by a positive beta-hCG level (cut-off beta-hCG 25 IU/mL) without the ultrasound evidence of a gestational sac. Miscarriage is defined as a spontaneous loss of pregnancy occurring before the 20th week of gestation. Live birth is defined as the birth of at least one infant after 24 weeks of gestation who survives for at least one month following either fresh or frozen embryo transfers after ovarian stimulation.

### 2.6. Statistical Analysis

We obtained our gene expression ratios by using a log-transformed approach, arc-transformed the proportions to ensure a Gaussian distribution, and analyzed them using GraphPad Prism version 9.0 for Windows (GraphPad Software, Boston, MA, USA, www.graphpad.com(accessed on 12 April 2025)). The reference expression levels of the genes of interest (GOI), namely, *HAS2*, *PTGS2*, and *GREM1*, were normalized to 0 by using RG (*RR18S* and *GAPDH*) as reference genes. Results are presented according to their distribution, specifically median ± interquartile range (IQR). We used a two-tailed Student’s t-test to compare the parameter of interest, with a *p*-value less than 0.05 considered statistically significant. To examine relationships among the skewed data, we conducted nonparametric correlation analyses, including a Kruskal–Wallis test, Spearman correlation test, Wilcoxon Signed-Rank test, and Mann–Whitney test. In addition, we assessed categorical associations by using Fisher’s exact test. An overall result of a *p*-value lower than 0.05 is considered statistically significant.

## 3. Results

### 3.1. Demographic

Our study included at least 30 women, with 15 participants from the NOR and DOR cohorts. The two groups were similar in age, duration of infertility, follicular oocyte index (FOI), and preovulatory follicle count on trigger day (FORT). Women in the DOR cohort showed significantly lower AMH levels, AFC, number of follicles aspirated, and number of oocytes retrieved despite undergoing oocyte pick-up (OPU) on the same day as their NOR counterparts. The DOR cohort required lower doses of gonadotropins than the NOR cohort. Most participants in both groups experienced primary subfertility, with the majority of the NOR cohort attributing their condition to male factors. Endometriosis was the primary cause of infertility in the DOR cohort. Both groups were administered hCG as the primary trigger agent before OPU; however, only the DOR cohort utilized dual triggers as part of their trigger strategy (Table 1).

### 3.2. Oocyte Maturation Outcome and Oocyte Quality [2] Outcome

**Figure 8:**
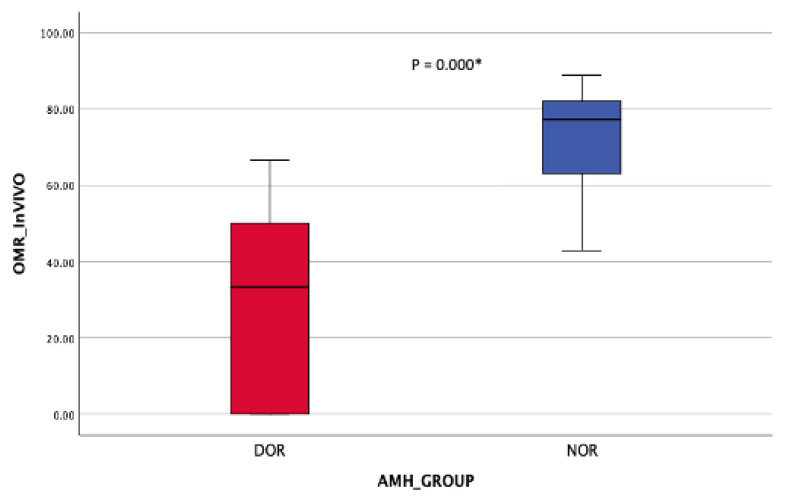
OMR in vivo (post-OR). Mann–Whitney Test * (significant).

**Figure 9:**
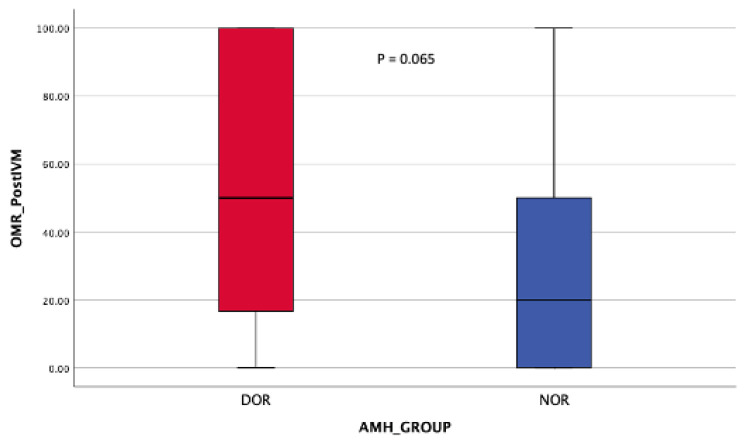
OMR post-r-IVM. Mann–Whitney Test.

Regarding the oocyte maturation outcome, the NOR cohort obtained significantly more MII oocytes post-OPU with a comparable number of MII oocytes post-IVM (Table 2). At least 70% in vivo oocyte maturation rates (OMR) were seen in the NOR cohort compared with 25% among DOR women (Figure 8). However, in vitro maturation (IVM) for OMR was higher in the DOR cohort, although the difference was not statistically significant (Figure 9). Improved OQ outcomes were seen in the DOR cohort, as more than 40% of poor OQ were observed in the NOR cohort, but the results were not statistically significant (Table 3).

### 3.3. Fertilization Outcome and Embryo Quality (EQ)

Significant MII oocytes may have undergone ICSI in the NOR cohort, with comparable fertilization rates (FR) observed in both cohorts. The cleavage stage was limited to the DOR cohort, while the blastocyst stage was specific to the NOR cohort (Table 4). However, the embryo quality (EQ) was similar in both cohorts, although more than 20% of the DOR cohort did not yield any embryos after ICSI. The NOR cohort exhibited poor EQ, while the DOR cohort produced a fair EQ that yielded improved outcomes; these differences were not statistically significant (Table 5).

### 3.4. Expression of HAS2, GREM1, and PTGS2 During Pre and Post IVM with OQ and EQ

**Figure 10:**
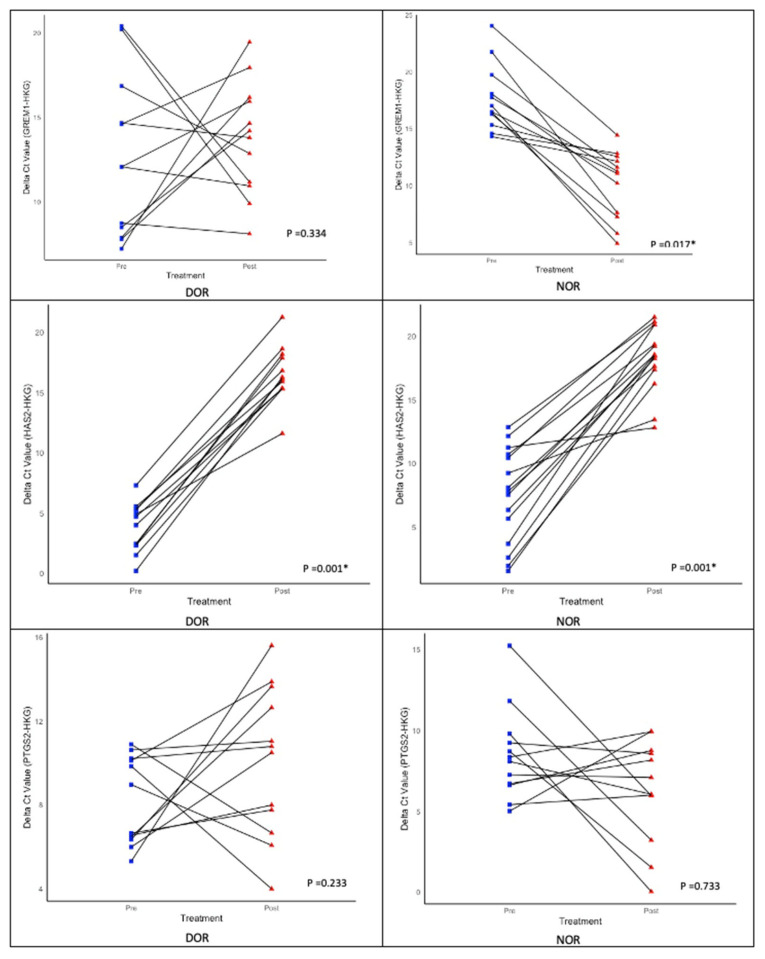
Expression of genes of interest (GOI)—HAS2, GREM1, and PTGS2—during pre- and post-r-IVM. Wilcoxon signed-rank test; * (significant).

Both cohorts demonstrated an upregulation of HAS2 following IVM, although this finding was not statistically significant (Table 6). PTGS2 and GREM1 showed significant downregulation in the normal ovarian reserve (NOR) cohort compared with the upregulated ovarian reserve [34] cohort, which also exhibited a notable decrease (Figure 10). When examining the relationship between age and anti-Müllerian hormone (AMH) levels, no correlation was observed with the fold change in all genes of interest (GOI), except for GREM1 (Table 7). Additionally, no correlation was found between fertility types and GOI (Table 8). However, GREM1 was significantly associated with all causes of subfertility (Table 9). The ovarian response rate (OMR) only correlated with GREM1 expression in the NOR cohort. Other GOIs were not associated with the DOR or NOR cohorts or different types of triggering agents (Table 10 and Table 11). In our cohort, HAS2, GREM1, and PTGS2 did not show any correlation with OQ and EQ (Table 12 and Table 13).

### 3.5. IVF/ICSI Outcome

In the rIVM-IVF cycle, all cohorts underwent frozen embryo transfer (FET). The NOR cohort had at least two embryos frozen compared with only one for the DOR cohort. All women in the NOR cohort underwent FET, which resulted in higher rates of failed implantation and a 33.3% clinical pregnancy rate. More than half of the women in the DOR cohort opted for FET, achieving a 40% clinical pregnancy rate but experiencing one failed implantation. At least one woman in the NOR cohort had a biochemical pregnancy compared with two women in the DOR cohort. Overall, the DOR cohort reported higher live birth rates, while the NOR cohort experienced more miscarriages (40% vs. 22.2%). However, these differences were not statistically significant (Table 14). Additionally, OQ did not correlate with pregnancy status or outcomes (Table 15), whereas EQ demonstrated a significant correlation (Table 16).

## 4. Discussion

In our cohort, we found that r-IVM benefits DOR women more compared with NOR women [5,29,43]. Most women in the DOR group were reported as elderly, aged 40 years and above [14]. However, to reduce age-related bias in our findings, we focused on women under 35 years. Otherwise, endometriosis is often the result of DOR, as shown in our cohort, mainly due to multiple operative interventions in the ovarian tissue (cystectomy, evisceration, and the pathogenesis itself), leading to DOR. In our cohort, most DOR women underwent a minimal stimulation protocol. This approach was selected because many of these women had previously used standard stimulation doses, leading to poor response outcomes. Therefore, to address the risk of repeated poor responses, our protocol focused on minimal stimulation for women with DOR. This approach resulted in a lower total dose of gonadotrophins than the NOR women. Despite obtaining fewer MII oocytes during OR in the DOR group, r-IVM yielded a comparable number of post-IVM MII oocytes that could be utilized in IVF cycles for both cohorts. Our study observed an oocyte maturation rate (OMR) of at least 50% in DOR women, which is consistent with previous research [5]. Most scholars agree that following stimulation and in vivo maturation, most oocytes progress optimally through internal signaling pathways needed for oocyte maturation outcomes [1,20,30]. However, a small percentage of oocytes may not progress properly because of signaling mutations or abnormal expression pathways, resulting in maturation failure in vivo.

Therefore, despite undergoing r-IVM, some oocytes remain immature, or, if they mature, their quality may still be compromised [1]. This phenomenon is frequently observed in NOR women, particularly those with PCOS. Oocytes from the NOR cohort are generally capable of maturing in vivo compared with those of women with DOR. However, in vivo maturation pathways may be disrupted due to suboptimal environment and stimulation, leading to a lower OMR [44]. By contrast, physiologically normal immature oocytes from the DOR cohort had improved outcomes with IVM, resulting in higher OMR post-IVM [14,27]. Nevertheless, we acknowledge that most oocytes following IVM exhibit poor oocyte quality [24], which contributes to limited implementation worldwide within the general infertility population. Interestingly, the OQ from the DOR cohort was better than that of the NOR cohort after r-IVM [5,45]. Nevertheless, OQ is multifactorial and is primarily influenced by age [46]. Since our cohort is relatively younger, OQ is more dependent on other factors, such as the causes of infertility and the direct effects of maturation. As previously mentioned, most oocytes from the DOR cohort are physiologically normal, suggesting that r-IVM does not interfere with overall quality [15,27,32]. Thus, our findings revealed that oocyte quality was rated as fair to good, which was better for women with DOR.

To date, OQ assessment has been determined through molecular analysis, microscopic assessment, and gene expression evaluation [39,47]. Various scoring systems have been published to evaluate OQ, and they primarily focus on total oocyte scoring (TOS) for accuracy [39,40]. The overall OQ can be predicted by utilizing factors such as cytoplasmic morphology, polar body size and shape, and size of the perivitelline space (PVS) (Figure 5). The complexity of OQ assessment has been acknowledged in recent reports. Techniques, such as Live Zona Imaging, Time-Lapse Imaging, and mitochondrial assessment, are currently employed [39]. Additionally, several genes have been identified as indicators of overall OQ, reflecting the microenvironment around individual oocytes. Key genes of interest include *GREM1*, *HAS2*, *PTGS2*, *growth differentiation factor 9 (GDF-9)*, *pentraxin 3 (PTX3)*, *epidermal growth factor receptor (EGFR)*, and specific *steroidogenic acute regulatory protein (STAR)* in cumulus cells (CCs), which are all related to oocyte developmental capacity and embryo morphology [48]. Our study evaluated gene expression to reflect OQ by using *HAS2*, *PTGS2*, and *GREM1* alongside two housekeeping genes (HKG), namely, *GAPDH* and *RR18S*. Our focus was on OQ following r-IVM. Therefore, we evaluated the expression of GOI before and after r-IVM. Our results showed that the DOR and NOR cohorts experienced an increase in *HAS2* expression. *PTGS2* and *GREM1* were downregulated in the NOR cohort compared with those in the DOR cohort (Figure 10). GOIs are involved in CCs to help control the microenvironment of developing oocytes for improved maturation and competency. In the CC level, *HAS2* coordinates the hyaluronan synthase pathways in the extracellular matrix and is essential for cell migration, tissue repair, and cell structure during oocyte development [47,48]. In both cohorts, we observed that *HAS2* levels increased following r-IVM, suggesting cellular repair occurs after this treatment. However, in the NOR cohort, other genes were not upregulated, despite r-IVM, which can be attributed to abnormalities in oocyte competency. Furthermore, PTGS2 regulates the cyclooxygenase-2 (COX-2) pathway linked to vascular endothelial growth factor (VEGF) signaling, which significantly affects overall oocyte development, quality, and nuclear maturation, crucial steps in developing mature oocytes.

Additionally, GREM1 influences transforming growth factor-beta (TGF-β) signaling, which controls cell growth, differentiation, and repair [47,49]. Our findings indicate that in the DOR cohort, there was an upregulation of both PTGS2 and GREM1, which correlates with their molecular functions—in improving VEGF, cell growth, differentiation, and repair—thereby enhancing overall oocyte maturation mainly in the DOR cohort. We observed a similar effect of these outcomes in our DOR cohort, resulting in improved oocyte quality, although this increase was not statistically significant. Nonetheless, a previous study concluded that GREM1 has a higher correlation with OM than the other two GOIs. Additionally, HAS2 was reduced in CCs after IVM, which contradicts our results, primarily due to different cohort profiles. However, other studies showed low PTGS2 after IVM, consistent with NOR women in our cohort.

GOIs significantly influence the microenvironment, affect oocyte maturation, and ensure improved fertilization and optimal embryo development, which is essentially oocyte competency. Our NOR cohort exhibited lower expression of GOIs, except for *HAS2*, indicating that the overall OQ post-IVM in these cases was of lower quality than that in DOR women. Many immature oocytes from NOR women were already abnormal, exhibiting lower competency and thus failing to mature in vivo. Consequently, despite r-IVM, the OQ remained similar, as r-IVM did not alter GOI expression and comparable fertilization rates. All GOIs influence 2PN fertilization with good sensitivity and specificity, which was not observed in our study. Moreover, age, AMH level, and subfertility type did not correlate with the expression of GOIs. None of the GOIs were linked to causes of infertility, except for *GREM1*. To our knowledge, *GREM1* plays a supportive role in cell repair within the CC microenvironment, and it was associated with a high oxidative stress environment, particularly in cases of infertility such as endometriosis, PCOS, and oncofertility [1,50].

We also found that GREM1 was correlated with oocyte maturation rates (OMR), especially among NOR women. Based on a previous study, the trigger agent does influence the signaling cascade involving both cumulus cells and the oocyte [51]. In addition to that, the expression studies on cumulus cells are valid because they provide a “window” into the health and developmental potential of the connected oocyte. This relies on the bidirectional communication within the cumulus-oocyte complex (COC) [52]. Thus, our study found that the expression of GREM1 was higher than that of other GOIs. Our study also revealed that in vivo maturation was associated with hCG triggers, whereas any trigger showed comparable results with IVM rates. These findings concur with previous evidence, indicating that hCG is the ultimate trigger for promoting oocyte maturation prior to OR [8,15]. While some evidence suggests that a dual trigger may benefit DOR women for improved maturation, we found no significant association [53]. Despite established evidence linking oocyte competency markers to good embryo development, we found no association between GOIs and all classes of OQ and EQ (poor, fair, or good) [47,54]. Our findings contradict established evidence primarily because our cohort included DOR and NOR women, and defective CCs contributed to lower GOI expression compared with optimal cohorts in existing research. Furthermore, our oocytes were cultured for maturation in r-IVM conditions, which involved suboptimal oocyte quality that could not mature in vivo. Consequently, GOIs did not correlate with OQ and EQ, as most of the oocytes can be abnormal. A small sample size could limit the generalizability of the outcomes.

In terms of fertilization, most women in the NOR group had male factor issues contributing to subfertility. However, the fertilization rates were higher in the NOR cohort, as they utilized more oocytes in ICSI cycles. Consequently, all embryos were cultured until day 5 for blastocyst formation. Our center opted for cleavage stage culture on day 3 because of low embryo numbers from insufficient oocyte utilization in the DOR cohort. Interestingly, the EQ appeared to be better in the DOR cohort, with most blastocysts from the NOR cohort being graded as having poor EQ. The compaction stage is crucial for determining good embryo quality; however, we examined oocytes from NOR women that failed to mature in vivo and underwent r-IVM, which, non-surprisingly, reflected negatively on their EQ [55]. Since all our cycles were IVF-IVM, they were ultimately subjected to FET cycles. In our study, all NOR women with usable embryos chose to proceed with FET, while only five DOR women opted for it. Our limited cohort suggested that DOR women had better outcomes following FET. At least 80% of DOR women achieved pregnancy, with a live birth rate of 75%, despite one case of miscarriage. Only 44% of pregnancies in the NOR cohort were positive, leading to a 50% live birth rate, with two cases resulting in miscarriage. This was not a pre-implantation genetic testing (PGT) cycle, so the euploid status of the embryos is unknown. The only predictive indicator identified was that the embryo quality was better in the DOR cohort. Our results indicated a strong association between pregnancy success and favorable embryo quality, regardless of the NOR or DOR status. However, as previously published, the implantation process is multifactorial, making it impossible to draw definitive conclusions [5,6,12,15]. Good embryo quality does not always guarantee successful pregnancy and live birth outcomes. Therefore, PGT and endometrial assessments should be used as standard evaluation because they correlate with future pregnancy success and live birth rates.

Our pregnancy status and outcomes were not associated with OQ but were correlated with EQ. Consistent with previously published data, most oocytes, regardless of quality, could fertilize (2PN), indicating that OQ was less significant than EQ in predicting pregnancy outcomes [1,39,42]. Therefore, OQ assessment is considered suboptimal and should not be considered an important parameter compared with EQ in predicting successful pregnancy outcomes. Our study’s strength is highlighting the overall outcomes for the younger cohort of women with DOR following r-IVM. We demonstrate an additional clinical indication for r-IVM to improve IVF outcomes. Furthermore, we evaluate the molecular evidence regarding the risk of jeopardizing the OQ following the r-IVM procedure by measuring the expression of GOIs before and after r-IVM. To ensure comprehensive observation, we tracked our cohort up to the point of delivery. Our study offers scientific insights and clinical recommendations for managing a complex group of women who experience poor outcomes, particularly those with DOR. Additionally, we provide evidence for the culture of r-IVM oocytes while keeping CCs intact to avoid complete denudation. This approach helps preserve the microenvironment necessary to support the IVM process.

However, our study has limitations, such as a small sample size, which prevents us from drawing definitive conclusions. Additionally, a single-center study was conducted, so we encourage future multicenter studies to re-evaluate the overall outcomes and provide better conclusions and recommendations. In future research, various molecular markers could be incorporated to explore additional potential gene evaluations in a non-invasive manner, such as examining CCs in DOR women following r-IVM. By utilizing the specific genes such as *STAR* genes, cellular staining via immunohistochemistry (IHC) or serum evaluation with interleukin markers via ELISA can adequately evaluate the potential inflammation effect of OQ, mainly the higher oxidation stress that can contribute to poor OQ and fertilization failure. Various experimental techniques can provide clear insights into the overall outcomes. Scholars should also explore the epigenetic outcome following r-IVM, which is crucial to ensure lifetime safety and acceptance. PGT-A can be used to assess the embryo’s chromosome outcome following r-IVM in DOR women. Following delivery, at least seven years post-delivery, children’s thriving outcomes should be evaluated and documented.

## 5. Conclusions

Our study provides new insights into the role of r-IVM in women with DOR. We observed improved *HAS2, PTG2*, and *GREM1* expressions in DOR women following r-IVM, indicating positive responses of COCs. This technique led to favorable OQ and EQ outcomes. Although we could not conclude the overall clinical outcome, we suggest that r-IVM can be offered to DOR women during the standard IVF cycle for improved fertility outcomes.

## Figures and Tables

**Table 1:** Baseline demographic (n = 30).

**Clinical Characteristic**	**NOR** **Median (IQR)**	**DOR** **Median (IQR)**	***p*-Value**
Age	32.0 (31.0–36.0)	33.0 (31.0–35.0)	0.785
Duration of infertility	5.0 (3.0–5.0)	5.0 (4.0–6.0)	0.362
AMH (ng/mL)	1.3 (1.2–1.3)	0.74 (0.13–1.0)	0.000 *
Antral follicles count (AFC)	12.0 (10.0–20.0)	4.0 (3.0–5.0)	0.000 *
Dose of gonadotropin	2925 (2925–2925)	1575 (1050–1575)	0.000 *
Day of OPU	13.0 (12.0–14.0)	14.0 (13.0–15.0)	0.026 *
No. of follicles aspirated	12.0 (9.0–14.0)	3.0 (2.0–4.0)	0.000 *
No. of retrieved oocytes	11.0 (8.0–16.0)	2.0 (2.0–3.0)	0.000 *
Follicular oocyte index (FOI)	80.0 (73.3–90.0)	75.0 (60.0–100.0)	0.337
Preovulatory follicle count on trigger day (FORT)	90.0 (88.9–91.7)	75.0 (66.7–100.0)	0.367
**Type of Subfertility**	**NOR** **n (%)**	**DOR** **n (%)**	***p*** **-Value**
Primary	12 (80)	13 (86.7)	1.000
Secondary	3 (20)	2 (13.3)	
**Causes on Infertility**	**NOR** **n (%)**	**DOR** **n (%)**	***p*** **-Value**
Male factor	5 (33.3)	1 (6.7)	0.036 ^#^
PCOS	4 (26.7)	0 (0)	
Adenomyosis	0 (0)	1 (6.7)	
Endometriosis	3 (20.0)	5 (33.3)	
Tubal factor	2 (13.3)	1 (6.7)	
Unexplained	1 (6.7)	3 (20.0)	
Oncofertility	0 (0)	4 (26.7)	
**Trigger Agent**	**NOR** **n (%)**	**DOR** **n (%)**	***p*** **-Value**
hCG	9 (60.0)	8 (53.3)	0.008 ^#^
Decapeptide	6 (40.0)	1 (6.7)	
Dual Trigger	0 (0)	6 (40.0)	

Mann–Whitney Test * (significant), Fisher’s exact test ^#^ (significant).

**Table 2:** Oocytes maturation outcome.

Oocytes Outcome	NOR Median (IQR)	DOR Median (IQR)	*p*-Value
No of MII	8.0 (6.5–11.5)	1.0 (0–1.0)	0.000 *
No of MI	2.0 (2.0–3.0)	1.0 (1.0–2.0)	0.003 *
No of GV	0 (0–1.0)	0 (0–0.5)	0.552
No of abnormal oocytes	0 (0–1.5)	0 (0)	0.143
No of MII post IVM	1.0 (0–1.5)	1.0 (0.5–1.0)	0.910
No of MI post IVM	2.0 (1.0–2.0)	1.0 (0–1.0)	0.001 *
No of GV post IVM	0 (0)	0 (0)	0.261
In vivo maturity rate	77.3 (66.7–79.5)	25.0 (0–50.0)	0.000 *
In vitro maturation rate	28.6 (0–45.0)	50.0 (16.7–58.3)	0.065

Mann–Whitney Test * (significant).

**Table 3:** Oocytes quality.

Embryo Quality Outcome	NOR n (%)	DOR n (%)	*p*-Value
Good	2 (13.3)	1 (6.7)	0.341
Fair	6 (40.0)	10 (66.7)	
Poor	7 (46.7)	4 (26.7)	

Fisher’s exact test.

**Table 4:** Fertilization outcome.

Fertilization Outcome	NOR Median (IQR)	DOR Median (IQR)	*p*-Value
No. of ICSI	9.0 (7–11.5)	1.0 (1.0–2.0)	0.000 *
No. of fertilization (2PN)	7.0 (5.0–9.0)	1.0 (1.0–1.0)	0.000 *
Fertilization Rate	72.7 (70.7–81.7)	66.7 (50.0–100.0)	0.866
Cleavage stage (D3)	0 (0)	1.0 (0.5–1.0)	0.708
Blastocyst stage (D5)	3.0 (0–4.0)	0 (0)	0.001 *

Mann–Whitney Test * (significant).

**Table 5:** Embryo quality.

Embryo Quality Outcome	NOR n (%)	DOR n (%)	*p*-Value
Good	2 (13.3)	1 (6.7)	0.06
Fair	6 (40.0)	8 (53.3)	
Poor	7 (46.7)	2 (13.3)	
N/A (no embryo yield)	0 (0)	4 (26.7)	

Fisher’s exact test.

**Table 6:** Genes of interest (GOI) expression (ΔΔCT)—pre- and post-IVM and fold change (FC) of HAS2, GREM1, and PTGS2.

AMH Group	Pre-IVM (ΔΔCT)	Post-IVM (ΔΔCT)	Z-Statistic	*p*-Value	Fold Change (FC)	*p*-Value
	GOI Expression Median (IQR)	GOI Expression Median (IQR)			GOI Expression Median (IQR)	
NOR	HAS2 0.36 (−3.76–3.24)	HAS2 10.47 (8.61–14.3)	−3.351	0.001 *	HAS2 0.001(0–0.003)	0.071
	GREM1 −0.73 (−2.63–2.51)	GREM1 −7.85 (−10.7–2.16)	−2.385	0.017 *	GREM1 231.5 (4.48–1624.9)	0.001 *
	PTGS2 0.09 (−1.65–1.53)	PTGS2 −0.99 (−6.90–2.32)	−0.341	0.733	PTGS2 1.99 (0.200–119.6)	0.049 *
DOR	HAS2 −0.07 (−1.91–272)	HAS2 13.78 (11.36–15.82)	−3.408	0.001 *	HAS2 0.0001 (0–0.00004)	0.071
	GREM1 −2.18 (−3.56–3.75)	GREM1 3.89 (−1.13–9.04)	−1.193	0.334	GREM1 0.068 (0.01–2.197)	0.001 *
	PTGS2 −0.07 (−1.30–3.50)	PTGS2 1.45 (0.42–7.30)	−0.966	0.233	PTGS2 0.366 (0.006–0.749)	0.049 *

Wilcoxon Signed Ranks Test; * (significant).

**Table 7:** The correlation between age, AMH level, and fold change (FC) of HAS2, GREM1, and PTGS2.

Parameter	GOI Expression (FC) Median (IQR)	r_s_	*p*-Value
Age 33.0 (31.75–36.00)	HAS2 0.0002 (0.00002–0.0009)	0.093	0.624
	GREM1 3.9395 (0.0554–540.65)	−0.040	0.834
	PTGS2 0.5053 (0.0481–7.448)	−0.083	0.662
AMH Level 1.18 (0.723–1.30)	HAS2 0.0002 (0.00002–0.0009)	0.342	0.065
	GREM1 3.9395 (0.0554–540.65)	0.604	0.000 *
	PTGS2 0.5053 (0.0481–7.448)	0.257	0.171

Spearman correlation test, r_s_ = Correlation Coefficient * (significant).

**Table 8:** The association between type of subfertility and causes of fertility with fold change (FC) of HAS2, GREM1, and PTGS2.

Type of Subfertility	GOI Expression (FC) Median (IQR)	X^2^ Statistic [37]	*p*-Value
Primary	HAS2 0.00014 (0.00002–0.000945)	0.007 (1)	0.933
	GREM1 3.398 (0.0433–696.289)	0.131 (1)	0.718
	PTGS2 0.5458 (0.0811–7.459)	0.410 (1)	0.522
Secondary	HAS2 0.0003 (0.000033- 0.0276)	0.007 (1)	0.933
	GREM1 90.431 (1.0976–374.714)	0.131 (1)	0.718
	PTGS2 0.0494 (0.0253–60.154)	0.410 (1)	0.522

Kruskal–Wallis Test.

**Table 9:** The association between causes of fertility and causes of fertility with fold change (FC) of HAS2, GREM1, and PTGS2.

Causes of Infertility	GOI Expression (FC) Median (IQR)	X^2^ Statistic [37]	*p*-Value
Unexplained	HAS2 0.00004 (0.000015–0.00022)	9.694 (6)	0.138
	GREM1 0.915 (0.00228–68.28)	14.811 (6)	0.022 *
	PTGS2 0.0591 (0.117–0.5873)	5.517 (6)	0.479
PCOS	HAS2 0.0019 (0.00028–0.0031)	9.694 (6)	0.138
	GREM1 530.59 (101.39–8088.13)	14.811 (6)	0.022 *
	PTGS2 4.734 (0.563–442.48)	5.517 (6)	0.479
Endometriosis	HAS2 0.000117 (0.000023–0.00073)	9.694 (6)	0.138
	GREM1 0.1021 (0.00618–12.057)	14.811 (6)	0.022 *
	PTGS2 0.6075 (0.5291–14.215)	5.517 (6)	0.479
Tubal Factor	HAS2 0.00031 (0.00005–0.00031)	9.694 (6)	0.138
	GREM1 231.472 (3.3980–231.472)	14.811 (6)	0.022 *
	PTGS2 0.3662 (0.492–0.3662)	5.517 (6)	0.479
Oncofertility	HAS2 0.00004 (0.000006–0.000071)	9.694 (6)	0.138
	GREM1 0.0505 (0.00084–1.1874)	14.811 (6)	0.022 *
	PTGS2 0.2394 (0.0056–43.46)	5.517 (6)	0.479
Male Factor	HAS2 0.000724 (0.000268–0.1269)	9.694 (6)	0.138
	GREM1 262.391 (3.907–2316.68)	14.811 (6)	0.022 *
	PTGS2 2.2513 (0.7914–248.205)	5.517 (6)	0.479

Kruskal–Wallis Test * (significant).

**Table 10:** The correlation between in vitro oocytes maturation rate (OMR) and fold change (FC) of HAS2, GREM1, and PTGS2.

AMH Group	GOI Expression (FC) Median (IQR)	r_s_	*p*-Value
NOR 28.57 (0–50)	HAS2 0.00071 (0.000051–0.0025)	0.096	0.733
	GREM1 231.473 (4.481–1624.87)	0.578	0.024 *
	PTGS2 1.988 (0.2005–119.55)	0.486	0.067
DOR 50 (0–66.67)	HAS2 0.0000713 (0.0000173–0.00433)	−0.015	0.958
	GREM1 0.0676 (0.00190–2.187)	0.049	0.863
	PTGS2 0.3662 (0.0064–0.74863)	0.432	0.108

Spearman’s correlation test, r_s_ = Correlation Coefficient * (significant).

**Table 11:** The association between type of trigger with in vitro maturation rate and in vivo maturation rate.

Type of Trigger	In Vivo Maturation Rates	*p*-Value	In Vitro Maturation Rates	*p*-Value
hCG	66.67 (29.17–76.39)	0.006 *	50.00(0–50.00)	0.695
Decapeptyl	77.27 (56.25–81.25)		28.57 (0–40.00)	
Dual Trigger	0 (0–37.5)		50.00 (0–62.50)	

Kruskal–Wallis Test * (significant).

**Table 12:** Correlation of oocytes quality [24] with fold change (FC) of HAS2, GREM1, and PTGS2.

Oocytes Quality [24]	GOI Expression (FC) Median (IQR)	X^2^ Statistic [37]	*p*-Value
Poor	HAS2 0.00031 (0.0000173–0.00326)	0.962 (2)	0.618
	GREM1 3.39 (0.0978–277.31)	1.477 (2)	0.478
	PTGS2 0.3661 (0.014–4.071)	1.396 (2)	0.498
Fair	HAS2 0.0000830 (0.0000293–0.000568)	0.962 (2)	0.618
	GREM1 2.004 (0.0071–586.024)	1.477 (2)	0.478
	PTGS2 0.505 (0.078–7.47)	1.396 (2)	0.498
Good	HAS2 0.000758 (0.000051–0.000758)	0.962 (2)	0.618
	GREM1 231.472 (16.00–231.472)	1.477 (2)	0.478
	PTGS2 18.70 (0.0494–18.70)	1.396 (2)	0.498

Kruskal–Wallis Test.

**Table 13:** Correlation of embryo quality (EQ) with fold change (FC) of HAS2, GREM1, and PTGS2.

Embryo Quality	GOI Expression (FC) Median (IQR)	X^2^ Statistic [37]	*p*-Value
Poor	HAS2 0.000141 (0.000177–0.0012)	0.522 (2)	0.770
	GREM1 4.481 (0.178–1071.42)	0.930 (2)	0.628
	PTGS2 0.3700 (0.0389–60.154)	1.259 (2)	0.533
Fair	HAS2 0.000366 (0.0000171–0.00274)	0.522 (2)	0.770
	GREM1 5.112 (0.0072–780.693)	0.930 (2)	0.628
	PTGS2 0.6067 (0.147–5.509)	1.259 (2)	0.533
Good	HAS2 0.000715 (0.0000513–0.000715)	0.522 (2)	0.770
	GREM1 231.47 (16.00–231.47)	0.930 (2)	0.628
	PTGS2 18.70 (0.0494–18.70)	1.259 (2)	0.533

Kruskal–Wallis Test.

**Table 14:** IVF/ICSI outcome.

**Frozen Embryo Outcome**	**NOR** **Median (IQR)**	**DOR** **Median (IQR)**	***p*** **-Value**
Total Embryo Frozen	2.0 (0–2.5)	1.0 (0–1.0)	0.077
Total Embryo Use	2.0 (2.0–2.0)	1.0 (1.0–1.0)	0.016 *
**Frozen Embryo Transfer (FET)**	**NOR** **n (%)**	**DOR** **n (%)**	***p*** **-Value**
Yes	9 (60.0)	5 (33.3)	0.076 ^#^
No	0 (0)	4 (26.7)	
N/A	6 (40.0)	6 (40.0)	
**Pregnancy Status**	**NOR** **n (%)**	**DOR** **n (%)**	***p*** **-Value**
Failed	5 (55.6)	1 (20.0)	0.300 ^#^
Biochemical	1 (11.1)	2 (40.0)	
Clinical	3 (33.3)	2 (40.0)	
**Pregnancy Outcome**	**NOR** **n (%)**	**DOR** **n (%)**	***p*** **-Value**
Miscarriages	2 (22.2)	2 (40.0)	0.766 ^#^
Live Birth	2 (22.2)	2 (40.0)	

Mann–Whitney Test * (significant), Fisher’s exact test ^#^.

**Table 15:** The association between oocyte quality with pregnancy status and outcome.

**Pregnancy Status**	**Oocytes Quality**			***p*** **-Value**
	**Poor n (%)**	**Fair**	**Good**	
Failed	1 (9.1)	4 (25)	1 (33.3)	0.321
Biochemical	1 (9.1)	1 (6.3)	0 (0)	
Clinical	2 (18.2)	2 (12.5)	2 (66.7)	
**Pregnancy Outcome**	**Oocytes Quality**			***p*** **-Value**
	**Poor n (%)**	**Fair**	**Good**	
Miscarriage	2 (18.2)	1 (6.3)	0 (0)	0.131
Live Birth	1 (9.1)	2 (12.5)	2 (66.7)	

Fisher’s exact test.

**Table 16:** The association between embryo quality with pregnancy status and outcome.

**Pregnancy Status**	**Embryo Quality**			***p*** **-Value**
	**Poor n (%)**	**Fair**	**Good**	
Failed	1 (11.1)	4 (28.6)	1 (33.3)	0.040 *
Biochemical	0 (0)	2 (14.3)	0 (0)	
Clinical	0 (0)	4 (28.6)	2 (66.7)	
**Pregnancy Outcome**	**Embryo Quality**			***p*** **-Value**
	**Poor n (%)**	**Fair**	**Good**	
Miscarriage	0 (0)	3 (21.4)	0 (0)	0.048 *
Live Birth	0 (0)	3 (21.4)	2 (66.7)	

Fisher’s exact test * (significant).

## Data Availability

The original contributions presented in this study are included in the article. Further inquiries can be directed to the corresponding author.

## Abbreviations

The following abbreviations are used in this manuscript:

ART	Artificial Reproductive Technique
cAMP	Cyclic Adenosine Monophosphate
COC	Cumulus-oocyte Complexes
DOR	Diminished Ovarian Reserve
FSH	Follicular Stimulating Hormone
GnRH	Gonadotropin-Releasing Hormone
GV	Germinal Vesicle
GVBD	Germinal Vesicle Breakdown
hCG	Human Chorionic Gonadotrophin
ICSI	Intra Cytoplasmic Sperm Injection
IVM	In Vitro Maturation
IQR	Interquartile Range
LH	Luteinizing Hormone
MI	Meiosis I
MII	Meiosis II
